# Fluctuating Hydrodynamics and Rare-Event Techniques Unveil the Mesoscale Physics of Boiling

**DOI:** 10.1017/jfm.2026.11639

**Published:** 2026-06-10

**Authors:** Andrea Bonanno, Filippo Occhioni, Marco Bussoletti, Mirko Gallo

**Affiliations:** 1 Dipartimento di Ingegneria Meccanica e Aerospaziale, Sapienza Università di Roma, Via Eudossiana 18, 00184 Roma Italy

**Keywords:** Bubble Dynamics, Nucleation Pathways, Wettability, Phase Change

## Abstract

Deciphering the incipient stages of phase change in superheated fluids—and their subsequent evolution—remains a major challenge, as it requires bridging microscopic nucleation with macroscopic bubble dynamics and heat transfer. This gap has long hindered predictive boiling models and limited progress in emerging thermal technologies. Here, we leverage large-scale simulations of fluctuating hydrodynamics, combined with a rare-event technique, to rationalise liquid**–**vapour transformation from nucleation to bubble growth. We quantify the statistics of nucleated bubbles and hydrodynamic fields, onset temperatures, and non-trivial wettability effects on the nucleation pathways. Despite operating at the mesoscale, the simulations recover microscopic nucleation signatures observed in atomistic studies, including the dependence of both the boiling onset temperature and the crossover between homogeneous and heterogeneous nucleation pathways on surface wettability. At larger scales, they reproduce the experimentally observed transition from an early exponential regime of bubble-contact areas to a post-critical power-law scaling. These findings establish a quantitative bridge between fluctuation-driven nucleation and emergent boiling dynamics across scales.

## 1 Introduction

Deciphering boiling in liquids has been pursued for decades through both experimental and theoretical analysis aimed at quantifying heat transfer and the dynamics of nucleated bubbles (Zhang et al. 2023). The motivation is clear: boiling is technologically pivotal across scales and sectors, from nuclear power plants (Wu et al. 2018) to next-generation thermal management in high-power-density microelectronics (Cho et al. 2016; Jones et al. 2018; Fan & Duan 2020). While the macroscopic behaviour of fully formed bubble populations is comparatively well understood (Georgoulas et al. 2015; Abbondanza et al. 2023a; Darshan et al. 2024; Abbondanza et al. 2024), what happens before bubbles appear—i.e., the incipient stages that trigger nucleation and set the subsequent dynamics—remains a compelling open question. This knowledge gap lies between atomistic and continuum descriptions of fluids (Lohse & Prosperetti 2016), precisely where phase change emerges and organises. In practice, the seemingly simple question “at what temperature does a liquid boil under constant pressure?” has a surprisingly complex answer. Experiments show that the onset temperature depends on both the imposed heat flux—faster heating leads to higher boiling temperatures—and on the surface chemistry, with hydrophobic substrates nucleating earlier than hydrophilic ones (Bourdon et al. 2015). These two trends can be rationalised physically: higher heat fluxes shorten the time available for rare nucleation events to occur, while hydrophilic walls increase the free-energy cost of bubble formation. However, such effects are very difficult to incorporate in conventional hydrodynamic (continuum) models in an operational and predictive manner, where the liquid to vapour phase change threshold is empirically identified, and the system is initialised with pre-existing embryos of the new phase (Chakraborty et al. 2024). These approaches have a limited predictability of boiling processes since they cannot establish the proper thermodynamic conditions at which phase change starts to take place. This atomistic feature can be captured by molecular dynamics (Menzl et al. 2016), but, for computational reasons, it leaves unexplored the mesoscale window (from nanometers to microns) where critical aspects of the transformation unfold. Mesoscale models, therefore, provide a natural framework for investigating these phenomena. Such models aim to capture the emergent behaviour originating at the microscopic (granular) scale and to transport it to larger scales through appropriately constructed free-energy functionals, which in turn form the basis for both deterministic and stochastic extensions out of equilibrium. On the deterministic side, diffuse-interface models of the van der Waals type have proven capable of accurately describing single-component two-phase systems (Anderson et al. 1998; Espanol 2001; Onuki 2005) as well as multicomponent mixtures (Liu et al. 2016; Mukherjee & Gomez 2022; Benilov 2023) across a wide range of problems, from the dynamics of cavitation bubbles (Hu et al. 2023; Magaletti et al. 2016, 2015b) to boiling (Laurila et al. 2012; Lombard et al. 2015) and condensation (Benilov 2024). Within the mesoscale modelling of multiphase systems, the Lattice Boltzmann method has also emerged as a powerful approach. Rooted in a kinetic description of fluids, it operates at an intermediate level between microscopic particle methods and macroscopic continuum equations (Swift et al. 1996; Benzi et al. 2006; Sbragaglia et al. 2009; Biferale et al. 2012; Falcucci et al. 2011). However, mesoscale models intended to describe phase change must also incorporate thermal fluctuations, which are ultimately responsible for the activated nature of the phase change process.

The theory of fluctuating hydrodynamics (FHD) provides a systematic framework to account for such effects. Originally proposed by Landau on phenomenological grounds, it was later given a proper microscopic foundation through coarse-graining procedures and projection-operator techniques à la Zwanzig (Zubarev & Morozov 1983; Español et al. 2009).

Over the years, this formalism has been extended to multiphase settings, including diffuse-interface models (Chaudhri et al. 2014; Gallo et al. 2018b) and phase-field formulations (Barker et al. 2023; Bell et al. 2025b,a), and has also been incorporated within Lattice Boltzmann approaches (Lulli et al. 2024). In this context, ensuring thermodynamic consistency between the prescribed free-energy functional, the dissipative structure of the equations, and the statistics of the stochastic forcing is essential in order to recover the correct Einstein**–**Boltzmann equilibrium distribution and, consequently, physically meaningful phase change pathways (Gallo et al. 2026). Multiphase FHD has been employed to investigate equilibrium and near-equilibrium properties of complex fluids, including the measurement of static structure factors and quench-induced spinodal decomposition from supercritical states (Chaudhri et al. 2014), as well as the estimation of critical exponents and phase separation (Lulli et al. 2024). Some of these authors have further applied this framework to cavitation phenomena, both in quiescent conditions (Gallo et al. 2018b, 2021) and in flowing liquids (Couette flow) (Gallo & Casciola 2024). In these earlier studies, nucleation was typically analysed by preparing the system in an initially metastable state, such as a liquid under tension, and allowing thermal fluctuations to trigger the transition following a quasi-equilibrium approach. In such settings, the metastable basin is well defined, and the activation process can be interpreted as a fluctuation-driven escape governed primarily by the underlying free-energy or quasi-potential landscape. In the context of boiling, however, the situation is intrinsically more complex. A liquid initially at saturation is subjected to rapid heating and therefore follows a strongly non-equilibrium path through a progressively increasing metastability. The evolution results from the competition between deterministic thermal forcing and stochastic fluctuations, with a strong and non-trivial coupling to heat transfer. In a recent study (Gallo et al. 2023), we demonstrated that stochastic thermal fluctuations, together with sparse defects on structured hydrophobic/hydrophilic surfaces, are essential to reproduce key macroscopic boiling observables. In particular, even small patches of hydrophobic material, when combined with thermal noise, can significantly accelerate boiling onset in a manner that is highly sensitive and difficult to control, possibly explaining the experimental ambiguity of boiling onset. These findings have opened the way towards a fluctuation-consistent description of boiling far from equilibrium and have highlighted the effectiveness of FHD as a predictive framework for nucleation processes under strongly non-equilibrium conditions.

Building on this foundation, in this work, we combine large-scale FHD direct simulations with rare-event analysis to chart the complete liquid-to-vapour transformation, from the earliest nucleation inception to bubble growth and interactions. By performing simulations over spatially extended domains, we access system sizes that are large enough to obtain robust spatial statistics. Our approach quantifies the temporal evolution of the probability distributions of temperature and density fields, revealing an unprecedented statistical description of non-equilibrium phase change. At the same time, it resolves the hydrodynamic fields that mediate bubble growth and interaction. We further gain access to valuable information that can be transferred to macroscopic models which do not explicitly resolve nucleation. This includes nucleation times, the formation of microscopic vapour films, and the statistical distribution of nucleation events. Finally, we focus on the role of wettability and characterise different nucleation pathways by computing the minimum free energy paths (MFEP) through rare-event techniques. Although these techniques rely on quasi-equilibrium constructions, they provide a powerful interpretative framework for the inherently non-equilibrium dynamics observed in boiling. The results align with both experimental observations and molecular dynamics benchmarks — across equilibrium features (such as wettability effects on boiling) and non-equilibrium dynamical scalings of boiling (onset dependence on heating rate and bubble dynamics) — thereby unifying diverse pieces of evidence into a coherent picture. Altogether, we advance a predictive framework for the incipient stages of phase change and their subsequent evolution, also providing a consistent energetic interpretation of nucleation and growth dynamics. By closing a crucial mesoscale gap, this enables principled multiscale strategies for boiling simulations and thermal-system design.

## 2 Methodology

### 2.1 Fluctuating Hydrodynamics (FHD)

The mesoscale model we employ to investigate the boiling process of a fluid initially at saturation (stable equilibrium) and subsequently heated at constant pressure into a metastable state combines the Landau**–**Lifshitz**–**Navier**–**Stokes equations with diffuse**–**interface thermodynamics (Gallo et al. 2021).

(2.1) $$\frac{\partial \rho }{\partial t}+\nabla \cdot (\rho \text{v})=0,$$

(2.2) $$\frac{\partial \rho \text{v}}{\partial t}+\text{∇}\cdot \left(\rho \text{v}⊗\text{v}\right)=\text{∇}\cdot \text{Σ}+\text{∇}\cdot \delta \text{Σ},$$

(2.3) $$\frac{\partial E}{\partial t}+\nabla \cdot (\text{v}E)=\nabla \cdot [(\Sigma +\delta \Sigma )\cdot \text{v}-q+\delta q].$$

*ρ* (**x**, *t*) is the mass density, ***ν*** (**x**, *t*) the velocity field, and *E* (**x**, *t*) the total energy density, defined as

(2.4) $$E=u_{b}(\rho ,T)+\frac{1}{2}\rho |\text{v}{|}^{2}+\frac{\lambda }{2}|\nabla \rho {|}^{2},$$

where *u*_*b*_ (*ρ, T*) is the bulk internal energy density, *T* (**x**, *t*) is the temperature, *λ* the capillary coefficient. In this framework, the balance equations for mass, momentum, and energy are augmented by stochastic fluxes that account for thermal fluctuations arising from the granular nature of matter, as well as by capillary contributions that modify the structure of both the stress tensor and the heat flux.

The deterministic stress tensor and energy flux, (Anderson et al. 1998; Magaletti et al. 2016; Giovangigli 2020), are

(2.5) $$\text{Σ}=\left[-p+\frac{\lambda }{2}{\left|\text{∇}\rho \right|}^{2}+\rho \text{∇}\cdot \left(\lambda \text{∇}\rho \right)\right]\text{I}-\lambda \text{∇}\rho ⊗\text{∇}\rho ++\eta_{1}\left(\text{∇}\text{v}+\text{∇}{\text{v}}^{T}\right)+\eta_{2}\text{∇}\cdot v\text{I},$$

(2.6) q=λρ∇ρ∇⋅v−k∇T,

$$\eta_{2}=\eta_{v}-\frac{2}{3}\eta_{1}$$

where *p* (*ρ, T*) is the pressure, **I** the identity matrix, *η*_1_ (*ρ, T*) and *η*_2_ (*ρ, T*) the viscosity coefficients, *k* (*ρ, T*) the thermal conductivity.

The stochastic fluxes (Chaudhri et al. 2014; Gallo et al. 2021) are Gaussian processes with zero mean and correlation:

(2.7) $$\langle \delta \Sigma (\hat{x},\hat{t})⊗\delta \Sigma^{†}(\tilde{x},\tilde{t})\rangle =Q^{\text{Σ}}\delta (\hat{x}-\tilde{x})\delta (\hat{t}-\tilde{t}),$$

(2.8) $$\langle \delta q(\hat{x},\hat{t})⊗\delta q^{†}(\tilde{x},\tilde{t})\rangle =Q^{q}\delta (\hat{x}-\tilde{x})\delta (\hat{t}-\tilde{t}),$$

where *δ*(**x**) and *δ*(*t*) are the spatial and temporal Dirac delta functions, respectively, and

$$\begin{array}{c} {{\text{Q}}^{\Sigma }}_{\alpha \beta v\eta }=2{\text{k}}_{B}T[\eta_{1}(\delta_{\alpha v}\delta_{\beta \eta }+\delta_{\alpha \eta }\delta_{\beta v})+\eta_{2}\delta_{\alpha \beta }\delta_{v\eta }],\\ {{\text{Q}}^{\text{q}}}_{\alpha \beta }=2{\text{k}}_{B}T^{2}k\delta_{\alpha \beta }, \end{array}$$

k_*B*_ is the Boltzmann constant, *δ*_*αβ*_ the Kronecker delta symbol. The noise amplitudes follow from the fluctuation**–**dissipation theorem, which ensures that the hydrodynamic description reproduces the equilibrium fluctuations of the underlying molecular system, consistent with the Einstein**–**Boltzmann distribution. Their intensity is directly determined by the transport coefficients, such as viscosity and thermal conductivity, reflecting the fundamental link between dissipation and spontaneous fluctuations in statistical mechanics (Kubo 1966). When in contact with a solid wall, the boundary condition for the mass density of the fluid is related to the fluid-solid wettability (Gallo et al. 2021),

(2.9) $$\lambda \nabla \rho \cdot \hat{\text{n}}=\text{cos}\phi \sqrt{2\lambda \left(\omega_{b}(\rho ,T)-\omega_{b}\left(\rho_{V}^{\text{sat}}\right)\right)},$$

with *ϕ* the Young contact angle, *ω*_*b*_ (*ρ, T*) = *f*_*b*_ − *ρ* (*∂ f*_*b*_ / *∂ρ*)_sat_ and *f*_*b*_ (*ρ, T*) the Landau and Helmholtz bulk free-energy densities, respectively, $\hat{\text{n}}$ the unit normal vector and the quantities labeled with sat refer to saturation values, see Section 3. The bulk energies *u*_*b*_, *f*_*b*_, *ω*_*b*_ and the pressure *p*, as well as the surface tension, are identified by a proper equation of state (Benilov 2020; Magaletti et al. 2021). These equations are very robust for describing the dynamics of bubbles. In fact, in the deterministic limit, they can reproduce the Rayleigh**–**Plesset behaviour and cavitation dynamics (Magaletti et al. 2015b,a; Abbondanza et al. 2023b), as well as the Hertz**–**Knudsen scaling (Benilov 2024) for the condensation of a planar interface. Beyond the deterministic limit, FHD provides a physically consistent framework that links thermal fluctuations to macroscopic fluid behaviour (Chaudhri et al. 2014; Donev et al. 2014; Gallo et al. 2018a; Bandak et al. 2022; Bell et al. 2022; Eyink & Jafari 2022; Magaletti et al. 2020; Barker et al. 2023; Eyink & Jafari 2024; Bussoletti et al. 2025, 2026; Teodori et al. 2025). It is therefore particularly well suited to investigate systems in which slow macroscopic (hydrodynamic) modes are intertwined with fast microscopic dynamics (atomistic fluctuations), as occurs in phase-change phenomena, especially under non-equilibrium conditions.

Here, the system of Eqs. (2.1, 2.2, 2.3) is closed through the van der Waals equation of state (EoS) relating the thermodynamic potentials, e.g. free energy densities or pressure, to the density and temperature fields. In particular, keeping the same notation, we rewrite both *p* and *u*_*b*_ in reduced variables by normalising pressure, temperature, and density by their corresponding critical values *p*_*c*_, *T*_*c*_, and *ρ*_*c*_, while the critical pressure normalises the internal energy density: *p* = 8*ρT*/(3 – *ρ*) − 3*ρ*^2^, *u*_*b*_ = 8*ρ* / (3*δ*) *T* − 3*ρ*^2^. Here *δ* = 0.112 is chosen to recover the specific heat for water.

### 2.2 FHD Simulations

**Figure 1:**
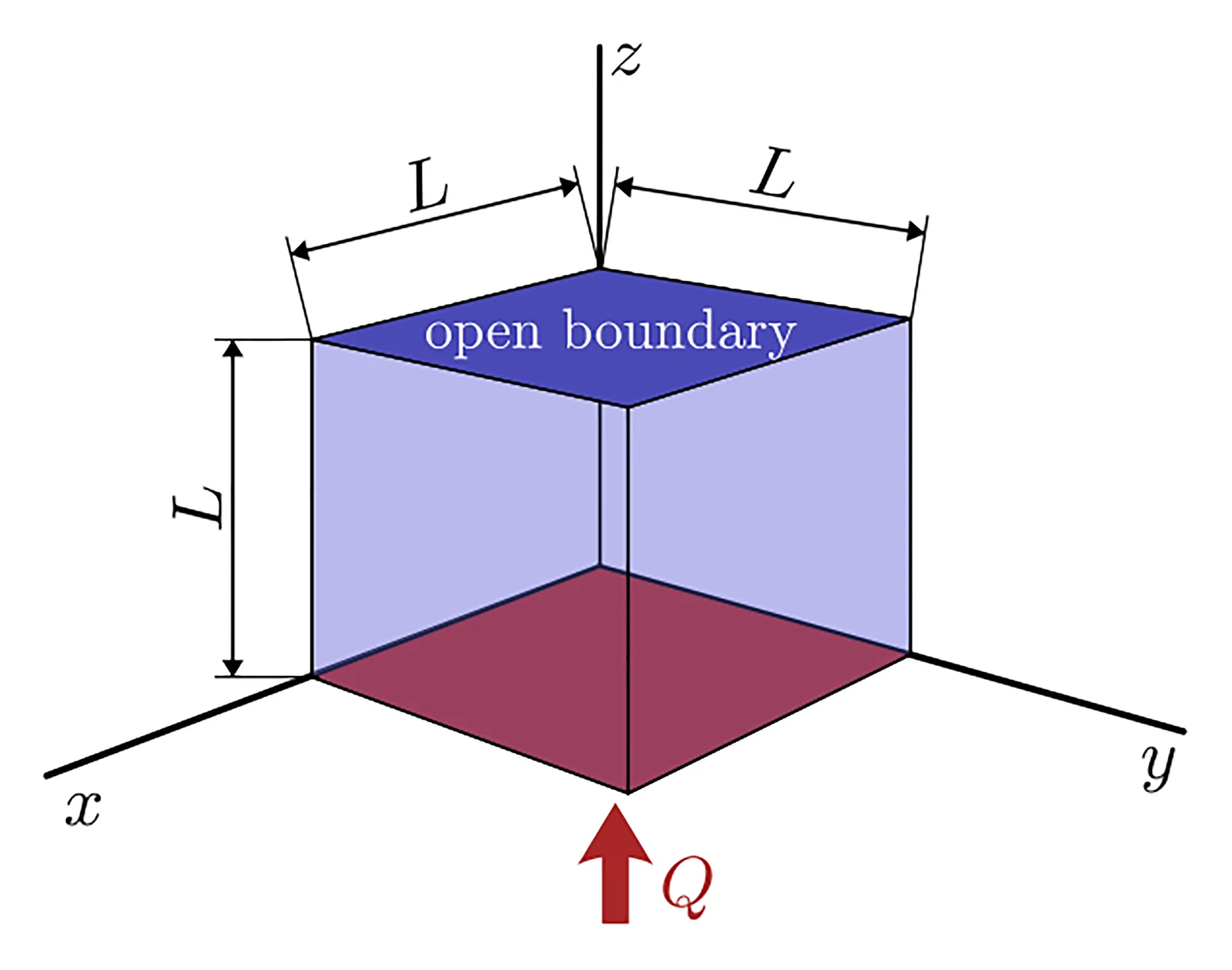
Sketch of the computational setup. The computational domain is a cubic box with side length *L*. A constant heat flux is imposed at the bottom, *z* = 0, solid wall together with the proper wettability conditions. At the top boundary, *z* = *L*, pressure and temperature are fixed to the saturation ones. Periodic conditions are imposed on the lateral boundaries of the domain.

The equations are integrated numerically with boundary conditions representative of the boiling configuration, as shown in Figure 1.

All simulations are performed in a cubic domain of side length *L*, discretised into uniform cubic cells of volume *Δ*^3^, so that *L*^3^ = *N*_*c*_ *Δ*^3^, where *N*_*c*_ is the total number of grid cells. In the present case *L* = 2.25*µ*m and *N*_*c*_ = 500^3^. The vertical coordinate **z** is oriented normal to the wall, with *z* = 0 corresponding to the heated surface in contact with the solid rigid wall and *z* = *L* to the top boundary of the domain. The other two directions, **x** and **y**, are parallel to the wall and periodic boundary conditions are imposed.

In pool boiling, the fluid far from the wall is maintained at saturation conditions; therefore, pressure and temperature are prescribed at the top boundary. On the wall, a constant heat flux is imposed, i.e. −**q·z** = *Q*. No-slip and impermeable boundary conditions are applied to the velocity field at the wall. The normal derivative of the density satisfies the solid–fluid boundary condition discussed above. At the top boundary (*z* = *L*), non-reflecting conditions are enforced to prevent spurious reflection of pressure waves (Poinsot & Lele 1992; Delgado-Buscalioni & Dejoan 2008). The numerical scheme follows (Gallo et al. 2018b) and employs a staggered finite differences discretisation with second-order spatial accuracy and a second-order Runge–Kutta time integrator. Due to the staggering of the discretisation, values of temperature and density at the wall — identified by the subscript “wall” in Section 3 — are extrapolated from the closest computational cell consistently with the imposed boundary conditions.

### 2.3 Minimum free-energy path (MFEP) and string method

The system of Eqs. (2.1, 2.2, 2.3) provides a stochastic hydrodynamic description of the non-equilibrium evolution of a heated phase-changing fluid. In this framework, bubble nucleation emerges as a rare event driven by thermal fluctuations and coupled to the underlying hydrodynamic fields. A natural first step towards rationalising this process is to adopt a purely thermodynamic viewpoint, in which nucleation is interpreted as a fluctuation that drives the system across a free-energy barrier separating the metastable liquid from the stable vapour phase. Within this quasi-static picture, the central quantity is the free-energy cost of forming a critical nucleus. This is identified by the saddle point of an appropriate constrained functional, thereby prescribing the transition pathway. However, boiling is inherently a dynamical, out-of-equilibrium phenomenon. In this context, rare transitions are more fundamentally described in terms of the probability of entire trajectories, which is governed by a dynamical action, for instance within the Freidlin–Wentzell or Onsager–Machlup formalism (Grafke et al. 2015; Zakine & Vanden-Eijnden 2023; Gallo et al. 2026). Extending such a large-deviation framework to the fully non-isothermal fluctuating hydrodynamics of Eqs. (2.1, 2.2, 2.3) remains, however, a formidable theoretical and computational challenge. For this reason, as a first step, we resort to a free-energy-based description which, despite its quasi-equilibrium character, is expected to retain key features of the incipient phase-change process. As will be shown in the Results section, this approach correctly predicts the existence of a critical nucleus, captures the scaling of nucleation barriers, and provides a consistent energetic interpretation of the transition pathways observed in our fluctuating hydrodynamics (FHD) simulations, as well as in recent molecular dynamics studies (Zou et al. 2018; Sullivan et al. 2025). Within this framework, the thermodynamic evolution of the heated liquid is interpreted as a sequence of quasi-equilibrium states of a Landau free-energy functional, each defined by contact with a reservoir imposing fixed temperature and chemical potential. In this grand-canonical setting, we evaluate, for each state, both the energetic cost associated with bubble formation and an approximation of the corresponding most probable transition pathway.

The Landau free energy of the mesoscale liquid–vapour system is modelled using the van der Waals squared-gradient approximation,

(2.10) $$\Omega [\rho ]=\int_{V}\left(f_{b}(\rho )+\frac{\lambda }{2}|\nabla \rho {|}^{2}-\mu_{\text{ext}}\rho \right)dV+\int_{A}f_{w}(\rho )dA,$$

where 𝒱 and 𝒜 indicate the domain and the bottom solid wall, respectively, *µ*_ext_ denotes the equilibrium chemical potential, and *f*_*w*_ (*ρ*) is the wall free-energy contribution,

(2.11) $$f_{w}(\rho )=-\text{cos}(\phi )\int_{\rho_{V}^{\text{sat}}}^{\rho }\sqrt{2\lambda \left(\omega_{b}(\rho ,T)-\omega_{b}\left(\rho_{V}^{\text{sat}}\right)\right)}+f_{w}\left(\rho_{V}^{\text{sat}}\right),$$

with $\rho_{V}^{\text{sat}}$ the vapour density at saturation (Gallo et al. 2021).

Although originally introduced on phenomenological grounds, this functional can now be derived within rigorous statistical-mechanical frameworks (Espanol 2001; Lutsko 2011). It is worth noting that the functional derivative of the energy in Eq. (2.10), *δΩ*/*δρ* = *µ*−*µ*_ext_, which represents the driving force of the system (i.e., the chemical potential difference), also appears in the non-equilibrium dynamics (Eqs. (2.1, 2.2, 2.3)); this is because the reversible part of the stress tensor

$$\Sigma_{\text{rev}}=\left[-p+\frac{\lambda }{2}|\nabla \rho {|}^{2}+\rho \nabla \cdot (\lambda \nabla \rho )\right]I-\lambda \nabla \rho ⊗\nabla \rho$$

obeys

$$\nabla \cdot \Sigma_{\text{rev}}=-\rho \nabla \left(\frac{\delta \Omega }{\delta \rho }\right),$$

at constant temperature, see (Abbondanza et al. 2024) for further details.

The metastable homogeneous liquid and the stable vapour phase correspond to local minima of *Ω* [*ρ*]. The transition between these states is represented by a path composed of a sequence of density fields. Assuming that the evolution is effectively dominated by the free-energy gradient, this sequence defines a minimum free-energy path (MFEP), which provides an approximation of the most probable transition pathway for a thermally fluctuating system (Gallo et al. 2025). In the present case, the MFEP describes the nucleation of a vapour bubble on a flat solid wall.

The MFEP is the sequence of fields *ρ*(*s*, **x)**, where *s* is a parametrisation variable that labels the configurations along the path satisfying

(2.12) $${\left(\frac{\delta \Omega }{\delta \rho }\right)}^{⊥}[\rho (s,x)]=0,$$

for the orthogonal projection of the functional derivative, and can be efficiently computed using the String Method (E et al. 2007).

### 2.4 MFEP simulations

Following the String Method, the path is discretised into a finite set of fields *ρ*^*s*^ (**x)**, referred to as images, where the index *s* labels the configurations along the path. These images evolve in a fictitious (pseudo-)time $\tilde{t}$ according to

(2.13) $$\frac{\partial \rho^{s}}{\partial \tilde{t}}=\mu_{eq}-\mu \left(\rho^{s}\right)+\lambda \nabla^{2}\rho^{s}.$$

The equations are discretised on a staggered grid in axisymmetric geometry and integrated in pseudo-time using a forward-Euler scheme. After each iteration, the images are redistributed along the path to enforce a uniform arclength parametrisation (E et al. 2007),

(2.14) $$\delta s=\|\rho^{s+1}(x)-\rho^{s}(x){\|}_{L^{2}}=\text{constant},$$

where $\|\cdot {\|}_{L^{2}}$ denotes the *L*^2^-norm. The evolution and redistribution steps are iterated until convergence, identified by the stationarity of the free-energy profile *Ω* (*s*). The resulting string connects the metastable liquid state at *s* = 0 to the vapour state at *s* = 1.

To minimise any bias associated with the initialisation of the path, two complementary simulation campaigns are performed. In the first, the string is initialised using MFEPs obtained at *ϕ* = 90°, corresponding to a heterogeneous nucleation bias. In the second, initial conditions are taken from MFEPs at *ϕ* = 0°, corresponding to a homogeneous nucleation bias. For each configuration, the solution with the lowest free-energy barrier is retained, and the nucleation mechanism is subsequently classified as homogeneous or heterogeneous based on the structure of the critical bubble. All simulations employ 200 images, a spatial resolution *Δ*^2^ = 1 on a domain of size *L*^2^ = 100^2^, and a pseudo-time step $\Delta \tilde{t}=0.01$. The initial stages of the path are further refined through Allen–Cahn relaxation (Bottacchiari et al. 2022, 2024).

### 2.5 Reference quantities for the numerical simulations

All quantities reported in the text and figures are dimensionless, scaled by the reduced van der Waals variables. Taking water as reference, the critical temperature, pressure, and density are *T*_*c*_ = 647 K, *p*_*c*_ = 22 MPa, and *ρ*_*c*_ = 196.8 kg/m^3^, respectively. The corresponding reference scales are: *L*_*R*_ = (*k*_*B*_ *T*_*c*_ / *p*_*c*_) ^1/3^ = 0.74 nm for length, *u*_*R*_ = (*p*_*c*_ / *ρ*_*c*_)^1/2^ = 334.8 m/s for velocity, *t*_*R*_ = *L*_*R*_ / *u*_*R*_ = 2.21 ps for time, and *q*_*R*_ = *p*_*c*_*u*_*R*_ = 7.56 GW/m^2^ for heat flux.

The capillary coefficient is set to *λ* = 5.3 × 10^−16^ m^7^ / (s^2^kg), ensuring the correct surface tension of water (*σ* = 0.072 N/m) at ambient conditions (*T* ~ 300 K). This choice yields a liquid–vapour interface thickness at ambient conditions of approximately *ϵ* ~ 1.3 nm, in agreement with experimental measurements (Caupin 2005). In the present case, the higher temperature *T* ≃ 0.95 − 0.96 leads to a broader, mean field interface, *ϵ* ~ 5.0 − 6.0 nm, and a reduced surface tension, *σ* ≃ 0.002 N/m. The transport properties, namely the viscosities *η*_1_, *η*_2_ (*ρ, T*) and the thermal conductivity *k* (*ρ, T*), are taken from the correlations of the International Association for the Properties of Water and Steam (IAPWS) (Kestin et al. 1984).

## 3 Results

The FHD equations presented in Section 2 were numerically solved to elucidate the boiling process of a liquid initially at saturation conditions and subsequently heated at constant pressure. All results are presented in non-dimensional form, as detailed in Section 2.

The initial liquid state corresponds to a (statistical) thermodynamic equilibrium, where thermal fluctuations are consistent with the Einstein–Boltzmann distribution (De Zarate & Sengers 2006). The average, initial values are $\langle \rho \rangle =\rho_{L}^{\text{sat}},\langle T\rangle =T_{\text{sat}}$, ⟨*T*⟩ = *T*_sat_ and ⟨**v**⟩ = **0**. As the liquid is progressively heated, it enters a metastable state in which the probability of vapour bubble nucleation increases with temperature, until the spinodal limit is reached, where phase change occurs spontaneously and without an energy barrier. The saturation conditions at fixed temperature *T*_sat_ are determined by imposing equality of the chemical potentials and of the pressures, $\mu \left(\rho_{L}^{\text{sat}},T_{\text{sat}}\right)=\mu \left(\rho_{V}^{\text{sat}},T_{\text{sat}}\right),p\left(\rho_{L}^{\text{sat}},T_{\text{sat}}\right)=p\left(\rho_{V}^{\text{sat}},T_{\text{sat}}\right),$ through the Van der Waals equation of state. These two conditions uniquely determine the liquid and vapour saturation densities $\rho_{L}^{\text{sat}}$ and $\rho_{V}^{\text{sat}}$. The spinodal temperature *T*_spin_ is defined as the temperature at which the pressure equals the saturation pressure, $p\left(\rho_{L}^{\text{spin}},T_{\text{spin}}\right)=p_{\text{sat}}\left(T_{\text{sat}}\right)$, where the spinodal considered here is that of the liquid phase, characterised by the condition ${\frac{\partial p}{\partial \rho }|}_{T}=0$. In the present case, the saturation and spinodal temperatures are *T*_sat_ = 0.95 and *T*_spin_ = 0.9625, respectively, while the saturation pressure is *p*_sat_ = 0.8119 and $\rho_{\text{sat}\;}^{L}=1.46471$. The intensity of the heat flux clearly sets the typical timescale for the appearance of the first bubbles. For the majority of the brute-force simulations presented here, we adopted a heat flux *Q* = 0.01 (in reduced units), which yields nucleation on numerically affordable timescales. Additionally, we varied *Q* to investigate the dependence of the onset nucleation temperature and time on the heating rate — an intrinsically non-equilibrium feature of considerable physical interest. By introducing the reduced temperature *Θ* = (*T* − *T*_sat_)/(*T*_spin_ − *T*_sat_), we have that *Θ* → 0 corresponds to a liquid saturation with zero probability of nucleating a bubble; on the other hand, *Θ* → 1 means spinodal conditions with spontaneous phase transition. This variable is a suitable descriptor of the process, as it provides a quantitative measure of liquid superheating up to the thermodynamic spinodal limit.

**Figure 2:**
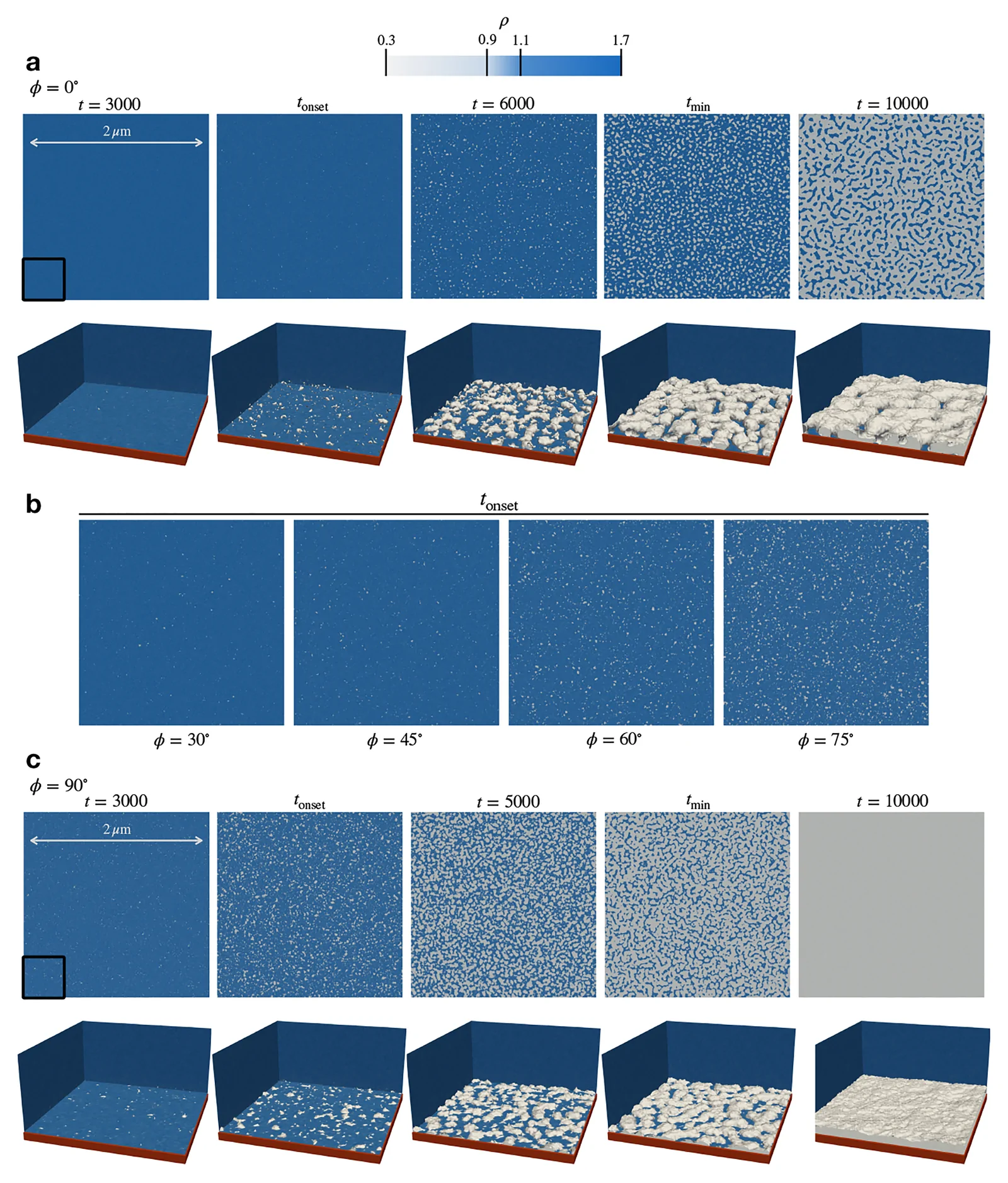
**Panel (a)**: In the top panel, simulation snapshots of vapour bubble nucleation and dynamics for a contact angle of *ϕ* = 0° are shown. Times are indicated above each frame. The upper contour plots display the density field at the wall, while the lower row presents a 3D zoom with isosurfaces at the critical density *ρ* (**x**, *t*) = 1. **Panel (b)**: The contour plots display the density field at the wall for different contact angles at the onset stage. **Panel (c)**: The panel reports the same quantities as Panel (a) for a contact angle of *ϕ* = 90°.

**Figure 3:**
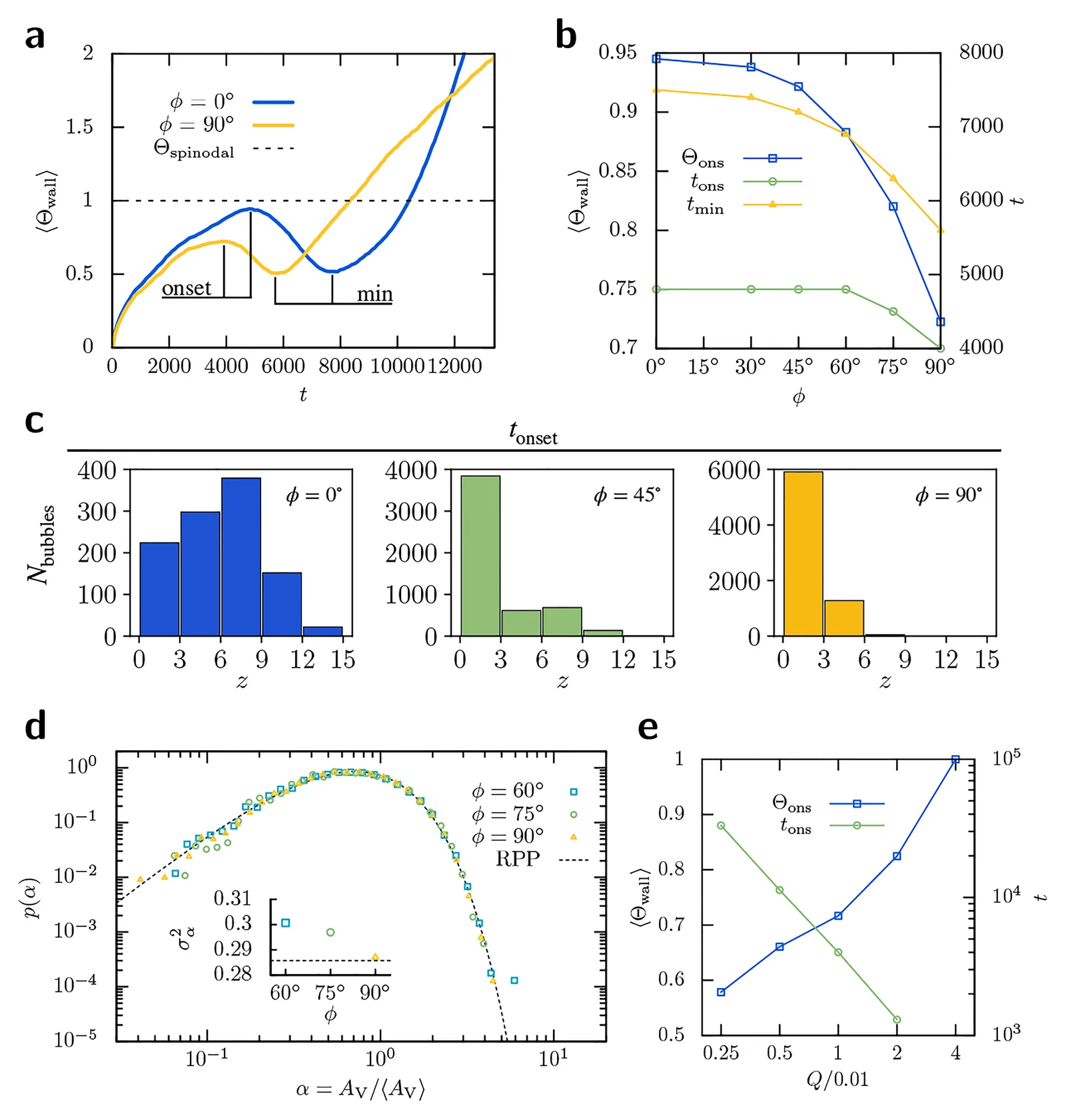
**Panel (a)**: Mean reduced wall temperature ⟨*Θ*_wall_⟩ as a function of time. Blue and yellow lines correspond to *ϕ* = 0° and *ϕ* = 90°, respectively. **Panel (b)**: On the left axis, reduced onset temperature $\Theta_{\text{ons}}={\langle \Theta_{\text{wall}}\rangle |}_{t_{\text{ons}}}$ as a function of the contact angle *ϕ* (blue line with squares). On the right axis, time to reach onset *t*_ons_ (green line with circles), and time to reach the post-onset minimum temperature *t*_min_ (yellow line with triangles). **Panel (c)**: Number of nucleated bubbles at the boiling onset as a function of the distance from the wall, *z*. From left to right, contact angles *ϕ* = 0°, 45°, and 90°. **Panel (d)**: Probability distribution of the normalized wall Voronoi cell areas *p*(*α*) at the boiling onset. Symbols refer to numerical simulations for different contact angles, while the dashed curve represents the PDF for an RPPP. The inset depicts the variance (symbols) and the comparison with the theoretical expectation (dashed line). **Panel (e)**: Reduced onset temperature *Θ*_ons_ as a function of the heat flux *Q* on the left axis (blue line with squares) and time to reach onset *t*_ons_ on the right axis (green line with circles); the data refer to the case *ϕ* = 90°.

Figure 2 shows representative snapshots of the numerical simulations for different contact angles. Panel (a) reports the case of a wall with contact angle *ϕ* = 0° (completely wettable), while Panel (c) corresponds to the neutral case with *ϕ* = 90°. The main difference lies in the nucleation mechanism. In the neutral case, nucleation initiates directly at the wall, following a more canonical boiling mechanism, whereas in the hydrophilic case, vapor bubbles nucleate within the liquid bulk, yet in proximity to the wall. In Panel (a) of Figure 2, the selected time instants illustrate the characteristic stages of the boiling process. The evolution of the wall temperature—and its link to the snapshots—is more clearly interpreted in Figure 3 (a). The stage at *t* = 3000 marks a typical time where bubbles form and grow, reaching the nucleation regime where the wall temperature attains its maximum, the boiling onset. Starting from this stage, the latent heat extraction exceeds the heat supplied by the surface, leading to a subsequent temperature drop toward its minimum at *t* = *t*_min_. After the phase transformation is completed and a continuous vapour film forms at the wall, the temperature rises again, as heat transfer becomes dominated by conduction across the vapour layer. Here, the minimum of the temperature curve corresponds to the instant at which the latent heat extracted by the phase change balances the externally imposed heat input. As anticipated, when *ϕ* = 0°, bubbles nucleate in the liquid close to the wall but not in direct contact with it; indeed, the wall-density contour plots show no coherent vapour structure beyond thermal fluctuations. Supercritical bubbles expand and later attach to the surface, where they continue to grow, coalesce, and eventually form a vapour nanofilm. This indicates the presence of a liquid nanolayer separating the bubbles from the solid wall. In fact, this liquid nanolayer was previously observed in molecular dynamics simulations (Zou et al. 2018; Sullivan et al. 2025), but to the best of our knowledge never reported in continuum boiling due to the intrinsic limitations of deterministic hydrodynamics, which cannot capture nucleation. Panel (b) of Figure 2 reports the density fields at the wall for intermediate contact angles (from 30° to 75°) at the boiling onset. One can observe that, as the contact angle decreases, the number of bubbles nucleating at the wall decreases as well, and the system transitions to different nucleation mechanisms, approaching homogeneous nucleation for highly hydrophilic wettability conditions. This peculiar behaviour will be further analysed using the string rare-event technique later on in this section.

Figure 3 (a) reports the temporal evolution of the mean reduced wall temperature ⟨*Θ*_wall_⟩ for *ϕ* = 0° (blue) and *ϕ* = 90° (yellow). The two characteristic points are highlighted: the first local maximum (onset) and the subsequent local minimum (post-onset minimum). The onset temperature *Θ*_ons_, defined as the mean reduced wall temperature at the onset, is shown in Figure 3 (b) (blue squares). It increases with decreasing contact angle but becomes nearly insensitive to wettability for *ϕ* ≤ 30°, where it almost plateaus at *Θ*_ons_ ≃ 0.95. The time to reach onset, *t*_ons_ (green circles), is also reported in Figure 3 (b). Contrary to the prediction of Classical Nucleation Theory (CNT), which prescribes a strictly increasing nucleation time with decreasing contact angle (Blander & Katz 1975), the simulations exhibit a plateau for *ϕ* ≤ 60°, approaching *t*_ons_ ≃ 4800. This suggests that, below a certain wettability threshold, nucleation occurs through a homogeneous mechanism, where bubbles first form near the wall—but not on it—and later reattach during growth, as also observed in macroscopic experiments (Zou et al. 2018), and as will be further discussed below within a quasi-equilibrium framework using rare-event arguments. The time to reach the post-onset minimum, *t*_min_ (yellow triangles), decreases with increasing *ϕ*, but similarly shows no marked dependence once *ϕ* ≤ 30°, further indicating that strong hydrophilicity suppresses the influence of wall wettability on both onset temperatures and nucleation times. Since the simulation covers sufficiently long times, it is possible to analyse statistics of bubble nucleation positions. It is worth emphasising that the wall is perfectly smooth, with no predefined nucleation sites; hence, nucleation events arise exclusively from stochastic fluctuations. A statistical characterisation of their space**–**time distribution is therefore informative, particularly for macroscale boiling models. Figure 3 (c) reports the number of bubbles nucleated at the onset temperature, *N*_bubbles_, as a function of the distance from the wall, *z*, for three different contact angles: *ϕ* = 0° (left), *ϕ* = 45° (center), and *ϕ* = 90° (right). As shown in the new panel, in the neutral-wall case (*ϕ* = 90°), essentially all nucleated bubbles are located very close to the wall. For the intermediate wettability case (*ϕ* = 45°), a lower number of nucleation events is observed, and the distribution becomes broader, extending toward larger distances *z* from the wall where nucleation events are also found. This trend becomes even more pronounced for the fully wetting case (*ϕ* = 0°), where nucleation preferentially occurs farther from the wall, and the total number of nucleated bubbles is even lower. While the presence of strong fluctuations makes it difficult to precisely reconstruct the earliest stages of nucleation solely from the bubble barycenter, the observed trends clearly indicate a progressive delocalization of nucleation events as wettability increases. A more refined statistical analysis, possibly combined with dedicated bubble-tracking algorithms, could further clarify these mechanisms in future work.

To characterise nucleation on the wall, we deploy Voronoi tessellations (Gallo & Casciola 2024) constructed from the centroids of nucleated bubbles. Figure 3 (d) reports the probability distribution of the normalised cell areas *α* = *A*_*V*_ / ⟨*A*_*V*_⟩, where *A*_*V*_ is the Voronoi area associated with bubble *i*. The analysis is performed at the onset for three different contact angles, 60°, 75°, and 90°, for which nucleation predominantly occurs at the wall. These distributions are compared with those of a Random Poisson Point Process (RPPP) (Snyder & Miller 2012), whose Probability Distribution Function (PDF), inferred from large-scale Monte Carlo simulations (Ferenc & Néda 2007), is $p(\alpha )=343/15\sqrt{7/(2\pi )}\alpha^{5/2}\exp(-7/2\alpha )$. Symbols denote the numerical simulations, while the dashed black line represents the RPPP prediction. The excellent agreement is confirmed by the variance (inset), which remains very close to the theoretical value *σ*_*α*_ = 0.286 (Ferenc & Néda 2007), for all the considered cases. This indicates that bubble nucleation on the wall is spatially uniform and uncorrelated, consistent with an RPPP, even up to the boiling onset. Accordingly, the expected number of nucleation events in a surface region *A*_wall_ during a time interval *T* is ⟨*N*⟩ = *J A*_wall_ *T*, with *J* the bubble nucleation rate. The probability of observing exactly *N* nucleation events follows *P* (*n* = *N*) = ⟨*N*⟩^*N*^ / *N*! exp (−⟨*N*⟩). Consequently, once the nucleation rate is available—whether obtained, for example, from fluctuating-hydrodynamics simulations or from a nucleation theory—it becomes possible to generate a statistically consistent ensemble of bubbles that captures the essential features of the otherwise elusive nucleation process. Panel (e) of Figure 3 shows the onset temperature (blue line with squares) as a function of the imposed heat flux intensity, together with the time required to reach it (green line with circles). Depending on the imposed heat flux intensity, the system remains in a metastable state for a time that increases as the heat flux decreases. Consequently, at higher heat fluxes, nucleation is expected to occur at higher temperatures, since the system has progressively less time to develop rare nucleation events. This behaviour highlights the intrinsically non-equilibrium nature of boiling.

**Figure 4:**
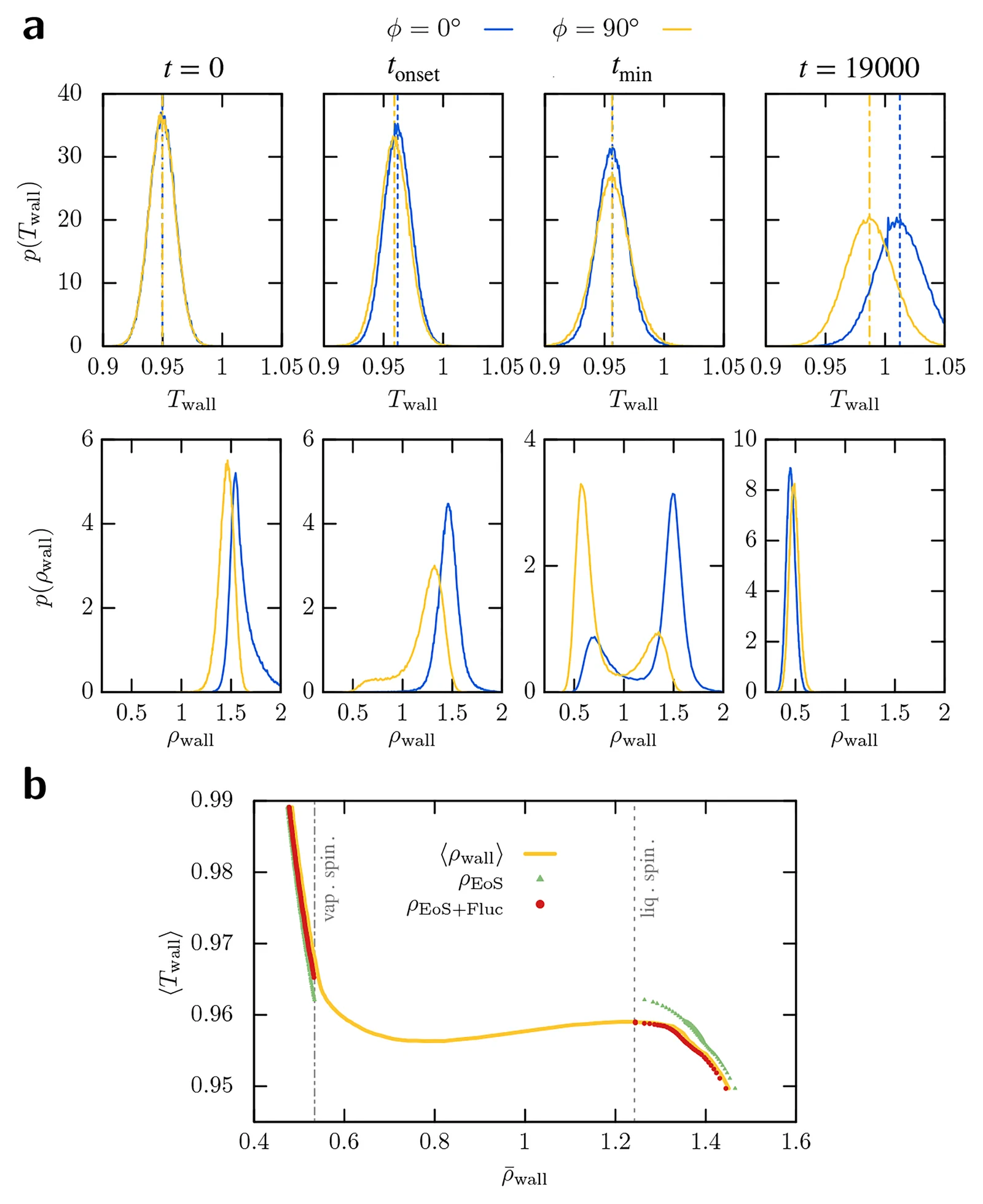
**Panel (a)**: Probability distributions of the wall temperature, *p* (*T*_wall_), top panel. Probability distributions of the wall density *p* (*ρ*_wall_), bottom panel. Different times during the boiling process are reported. Yellow curves correspond to *ϕ* = 90°, while blue curves correspond to *ϕ* = 0°. **Panel (b)**: Evolution of the mean wall temperature ⟨*T*_wall_⟩ as a function of the mean density ${\bar{\rho }}_{\text{wall}}$. The yellow curve corresponds to numerical simulations; the green triangles correspond to the density on the isobar *p* = *p*_sat_ without accounting for fluctuations (mean-field); the red circles include thermal fluctuation corrections.

Phase change is triggered by thermal fluctuations and mediated by the nonlinear hydrodynamics of the nucleated bubbles. A statistical description is therefore essential. Figure 4 (a) shows the probability distribution function (PDF) of the wall temperature (top) and the density (bottom). The blue line corresponds to the wetting condition with *ϕ* = 0°, while the yellow line refers to *ϕ* = 90°. At *t* = 0, the fluid is at equilibrium. The temperature distribution is Gaussian, with variance scaling as var(*T*) ~ *k*_*B*_*T*/(*ρ*_wall_*c*_*ν*_), with *k*_*B*_ the Boltzmann constant and *c*_*ν*_ the specific heat at constant volume. At later times, the PDFs shift to the right due to an increase in the mean wall temperature, and broaden as vapor formation enhances thermal fluctuations. For the density, a weak asymmetry is already visible at *t* = 0. This arises because, at nanometric scales, density fluctuations are sufficiently large to depart from the linear regime.

In general, rarefaction events would be more probable than compression, consistent with the scaling var(*ρ*) ~ *k*_*B*_*T ρ*^2^*β*_*T*_, where *β*_*T*_ = 1/(*ρ ∂ p*/*∂ ρ*) denotes the isothermal compressibility. Indeed, the compressibility of the liquid phase is significantly lower under mild compression than under mild rarefaction. However, the hydrophilicity of the wall at *ϕ* = 0° strongly favors compressed liquid states. As time progresses, nucleation leads to the emergence of heavy tails on the left side of the density PDFs. These tails are more pronounced for the case *ϕ* = 90°, where a lower activation barrier facilitates nucleation in terms of free energy and bubbles are nucleating only on the wall (see the discussion on nucleation energetics below). The process then evolves toward a quasi-bimodal distribution, reflecting the coexistence of liquid and vapor regions within the system. Eventually, once the transition to the vapor phase is complete, the PDFs revert to an approximately Gaussian shape. In Panel (b) of Figure 4, we report two macroscopic observables that provide an integrated view of the overall boiling process: the mean wall temperature ⟨*T*_wall_⟩ plotted against the mean wall density ${\bar{\rho }}_{\text{wall}\;}$, at fixed system pressure. In particular, ⟨*T*_wall_⟩ is the numerical average from the FHD results, while ${\bar{\rho }}_{\text{wall}\;}$ is derived in three different ways. The yellow curve represents the numerical results obtained from our FHD simulations, ⟨*ρ*_wall_⟩. The green curve shows the theoretical prediction, *ρ*_EoS_, based on the van der Waals equation of state, neglecting fluctuations. This amounts to solve *p*_sat_ = *p* (*ρ*_EoS_, ⟨*T*_wall_⟩). The red curve, instead, includes the fluctuation-induced correction proposed in the present work. The predicted mean wall density, *ρ*_EoS+Fluc_, is now given by solving

(3.1) $$\begin{array}{c} p_{\text{sat}\;}=\langle p_{\text{Fluc}\;}\rangle =p\left(\rho_{\text{EoS+Fluc}\;},\langle T_{\text{wall}\;}\rangle \right)+{\frac{1}{2}\frac{\partial^{2}p}{\partial \rho^{2}}|}_{\left(\rho_{\text{EoS+Fluc}\;},\langle T_{\text{wall}\;}\rangle \right)}\mathrm{var}\left(\rho_{\text{wall}\;}\right)\\ +{\frac{1}{2}\frac{\partial^{2}p}{\partial T^{2}}|}_{\left(\rho_{\text{EoS+Fluc}\;},\langle T_{\text{wall}\;}\rangle \right)}\mathrm{var}\left(T_{\text{wall}\;}\right), \end{array}$$

which demonstrates that accounting for fluctuations is essential even at the macroscopic level. The value of the field variances has been evaluated as proposed in (Gallo 2022), using *ρ*_EoS+Fluc_ as mean wall density. All theoretical curves terminate at their respective spinodal points, where the function loses its single-valued character.

To interpret the FHD simulations, the most probable nucleation pathways are evaluated under a quasi-equilibrium assumption, allowing estimation of the free-energy barrier *ΔΩ*^†^ from the MFEP, see Section 2.3.

We performed 208 distinct combinations of superheat and contact angle. Specifically, the superheat *Θ* is sampled in the range *Θ* = 0.2–0.95 with increments *ΔΘ* = 0.05, while the contact angle spans *ϕ* = 0°–90° with increments *Δϕ* = 7.5°. The resulting energy barriers are then interpolated using cubic splines to obtain smooth contour plots. Owing to the dual initialisation procedure described in Section 2.4, the total number of string simulations amounts to 384.

**Figure 5:**
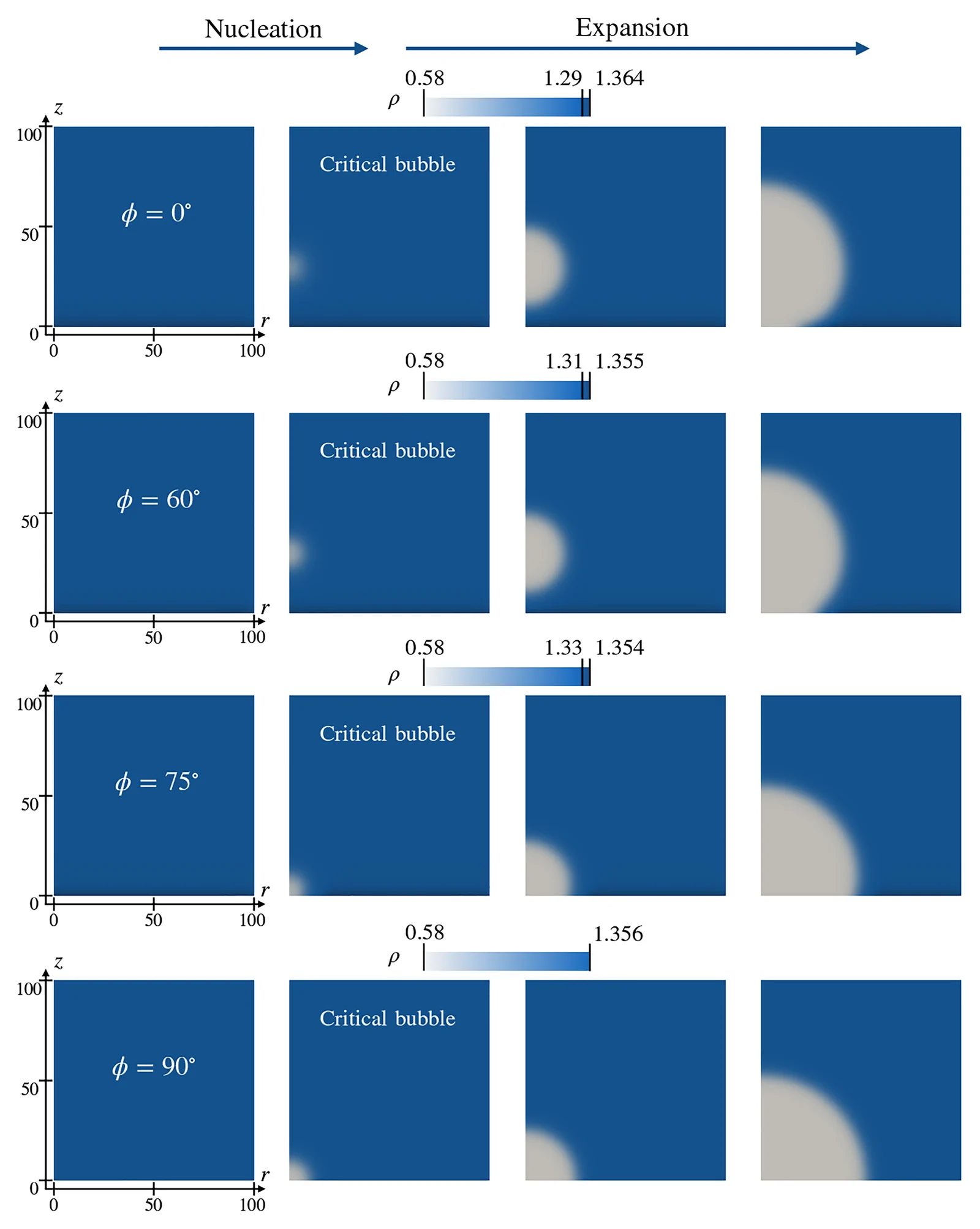
Density profiles along the transition path, showing pre-critical and critical configurations (first two panels from the left), followed by post-critical configurations (last two panels); different contact angles are reported. For *ϕ* = 0°, 60°, and 75°, the dark blue shade distinguishes the presence of the compressed liquid nanolayer.

**Figure 6:**
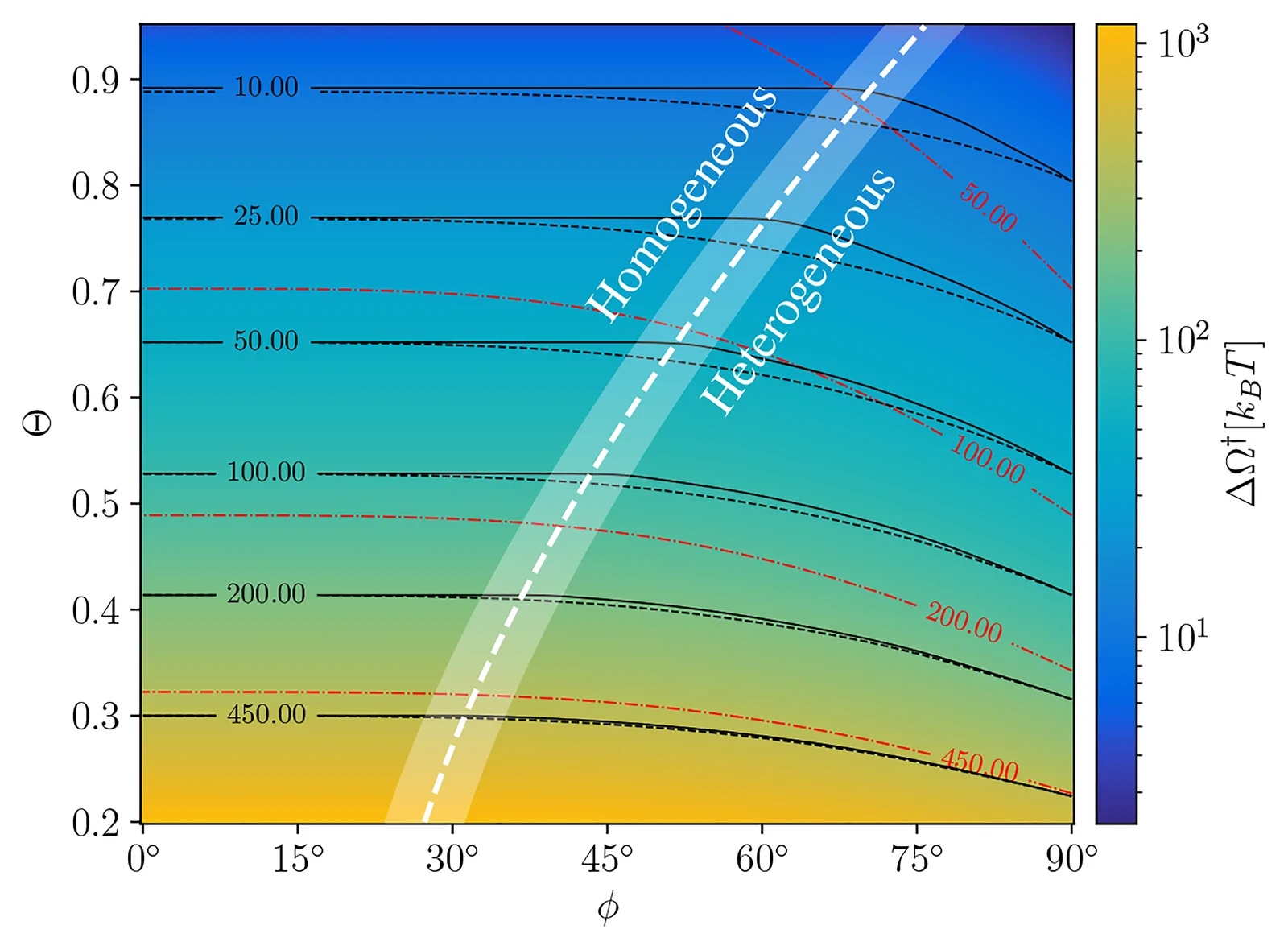
Normalised free-energy barrier *ΔΩ*^†^ as a function of reduced temperature *Θ* and contact angle *ϕ*. Solid lines represent iso-barrier contours predicted by the string method. Red dashed lines show classical nucleation theory (CNT) estimates. Black dashed lines are the CNT correction obtained by rescaling the homogeneous string barrier with the geometric function $\psi (\phi )=\frac{1}{4}{\left(1+\text{cos}\phi \right)}^{2}(2-\text{cos}\phi )$. The white line represents the demarcation boundary between the homogeneous and heterogeneous nucleation regimes, with the surrounding band indicating the uncertainty of the analysis in terms of the simulation discretisation *ΔΘ* and *Δϕ*.

The resulting MFEPs are reported in Figure 5. The simulations are performed at the onset conditions identified in the FHD simulations for each wettability, i.e. as if the system were statically and uniformly at the onset temperature as previously defined (see Figure 3). For *ϕ* = 0°, strong wall affinity leads to the formation of a liquid nanolayer, and nucleation occurs in the bulk rather than at the wall. This behaviour appears to persist up to contact angles of approximately 60°. In this regime, the critical bubble emerges close to, but not attached to, the surface, consistent with our FHD results (see Figure 2) and MD simulations (Zou et al. 2018; Sullivan et al. 2025); in later stages, bubbles expand and eventually attach to the wall. On the other hand, when *ϕ* = 90°, no nanolayer develops due to the boundary condition *∂ρ*/*∂n* = 0 at the wall, while at *ϕ* = 75° the effect remains weak and nucleation occurs on the wall. In Figure 6, we report the string nucleation free-energy barriers *ΔΩ*^†^, normalised by *k*_*B*_*T* for different contact angles and degrees of superheating. Iso-energetic curves are superimposed on the color map as solid black lines to facilitate interpretation. The red curves represent the prediction of (sharp-interface) CNT, which overestimates the barrier, as it does not account for curvature-dependent surface tension effects (e.g. Tolman corrections) and cannot capture the diffuse nature of the interface (Menzl et al. 2016). These effects are instead naturally included in diffuse-interface models (Gallo et al. 2025). According to CNT, the heterogeneous barrier is $\Delta \Omega_{\text{CNT}}^{†}=\psi (\phi )\Delta \Omega_{\text{CNT}}^{\text{†Hom}}$, with $\psi (\phi )=\frac{1}{4}{\left(1+\text{cos}\phi \right)}^{2}(2-\text{cos}\phi )$, which is strictly decreasing with *ϕ* and approaches the homogeneous limit as *ϕ* → 0. CNT also predicts a finite barrier as *Θ* → 1, a known limitation (Unger & Klein 1984). CNT predictions deteriorate as metastability increases, as interfacial effects become progressively more pronounced. The dashed black curves, instead, are obtained by rescaling the homogeneous barrier computed via the string method with the function *ψ* (*ϕ*). As shown, this scaling—indicative of wall-induced nucleation—works well at low metastability, *Θ* ≃ 0.2. However, at higher metastability, i.e. for larger values of *Θ* as in our simulations, clear deviations emerge and the nucleation mechanism changes. Notably, brute-force (molecular dynamics and FHD) simulations are typically performed in this regime. From this analysis, a transition region between different nucleation mechanisms can be identified (white dashed curve), with the surrounding shaded band indicating the uncertainty due to discretisation in *Δϕ* and *ΔΘ*. In particular, in the regime explored by our FHD simulations, where *Θ* ≳ 0.7, the predicted mechanism is homogeneous for contact angles *ϕ* ≲ 60°. This behaviour is consistent with the FHD results, where the time required to observe nucleation becomes approximately independent of *ϕ* for contact angles below about 60°, suggesting that homogeneous nucleation becomes the dominant mechanism in this regime (see Panel (b) of Figure 3). As such, nucleation times are expected to be governed by the most probable nucleation pathways. However, it is important to note that when the energy barriers associated with different nucleation pathways are comparable, as in the intermediate wettability regime, nucleation may occur via both mechanisms with different probabilities. These probabilities are not only controlled by the barrier heights, but also by configurational factors and the density of available nucleation sites. In particular, bulk nucleation scales with the system volume, whereas wall nucleation scales with the surface area, leading to different prefactors in the associated transition rates. This explains why both mechanisms are observed in the FHD simulations at intermediate wettabilities, as evident from Figure 3 (c). A more quantitative assessment would require rare-event sampling at finite temperature (i.e. in the presence of finite noise), in order to properly capture the competition between pathways. This is currently under investigation and will be the subject of future work.

The onset temperature, as defined in the dynamic simulations, depends not only on nucleation but also on heat transfer with the surrounding fluid and hydrodynamic interactions. Similarly, the formation of the vapour film is influenced by the same mechanisms, and both the onset temperature and the associated timescales retain a residual, albeit weaker, dependence on the contact angle even below 60°. For instance, bubbles tend to nucleate close to the wall and locally cool the nearby region, although not exactly at the wall. Therefore, the onset cannot be interpreted purely in terms of energetics.

**Figure 7:**
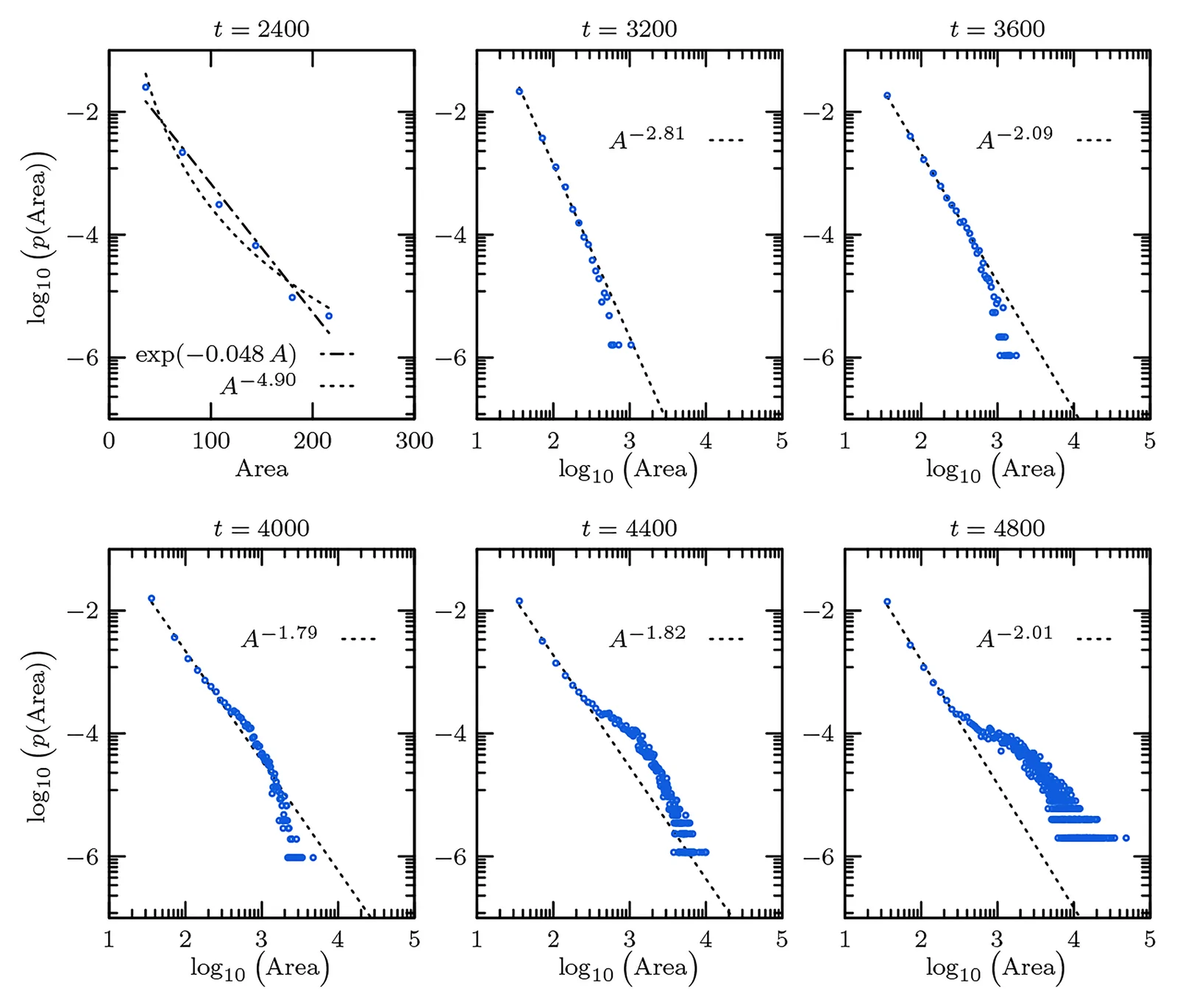
Probability distribution of bubble contact areas on the wall *p* (Area). Blue circles represent simulation data for *ϕ* = 90°, while dashed lines correspond to fitted PDFs. The simulation times at which the distributions are computed are indicated above each panel.

As a final analysis, the PDFs of the bubble**–**wall contact areas during the entire phase transition are examined in Figure 7. In the nucleation stage (top-left panel), the PDF exhibits an exponential tail. Indeed, the probability of observing a bubble of subcritical radius *R* scales as *p* (*R*) ~ exp (−*ΔΩ* (*R*)/ *k*_*B*_*T*), with *ΔΩ* ~ *R*^2^ (Menzl et al. 2016). After nucleation, the bubble growth dynamics becomes governed by hydrodynamic interactions and phase-change kinetics, and the PDFs develop scale-free behaviour characterised by power-law tails, *p* ~ *A*^−*γ*^. This emerges from collective amplification, coalescence, and merging events, reflecting the absence of a characteristic length scale during intermediate stages. The critical exponent *γ* evolves over time and remains consistent with those reported in macroscopic boiling experiments (Zhang et al. 2019). After the exponential regime, the first power-law stage yields exponents in the range *γ* ≃ −2.81 to −2.09. Beyond the boiling onset, the scaling becomes shallower, with an average exponent of *γ* ≃ −1.87, in good agreement with the experimentally reported post-critical values of *γ* ≃ − 1.85 and − 1.50 (Zhang et al. 2019). At later times, a noticeable bump emerges beyond the power-law regime, indicating the formation of large vapor clusters whose lateral extent becomes comparable to the system size, preceding film formation. Such behavior—also interpreted in (Zhang et al. 2019) as a percolation-driven transition—signals the onset of spatial connectivity and near-critical cluster coalescence. It is worth noting that, although the applied heat flux (*Q* = 0.01, ~ 70 MW / m^2^) lies well within the boiling crisis regime, the incipient nucleation stage still displays an exponential behavior with spatially uniform events. Because the system under consideration operates at the mesoscale (with dimensions of a few microns) and involves an ideally smooth wall, the observed scale-free dynamics cannot be attributed to surface roughness or chemical heterogeneities. These effects, while inevitable in macroscopic boiling experiments, are absent here. The emergence of scale-free behavior must therefore originate from the intrinsic multiphase hydrodynamics of the system itself. At the same time, the nucleation process—still experimentally inaccessible—retains a canonical exponential statistics, even under boiling-crisis conditions. This separation of behaviors indicates that supercritical boiling is characterized by an initial nucleation stage whose statistical properties are remarkably similar to those observed in pre-critical boiling, while the subsequent dynamics follows a markedly different, scale-free regime.

## 4 Discussion

This work combines high-performance computing and mesoscale fluctuating hydrodynamics to provide a comprehensive statistical picture of boiling as a thermally activated, intrinsically non-equilibrium process. In this framework, macroscopic observables emerge from the interplay between thermal fluctuations—responsible for triggering nucleation—and hydrodynamics, which governs bubble growth, interactions, and scaling laws. Despite operating at the mesoscale (≃ 10 *µ*m^3^), the simulations reproduce microscopic nucleation features—such as the formation of a liquid nanolayer as well as the dependence of the boiling onset and the dominant nucleation mechanism on wall wettability—that are typically observed only in molecular dynamics (Zou et al. 2018; Sullivan et al. 2025) and are inaccessible to classical continuum theories. These behaviours can still be interpreted within a rare-event framework based on free-energy landscapes. Moreover, the simulations recover the experimentally observed transition from an early exponential regime of contact areas to a post-critical power-law scaling, with critical exponents matching macroscopic measurements. This establishes a quantitative bridge between microscopic nucleation mechanisms and the emergent macroscopic boiling dynamics.

Beyond resolving fundamental questions, our results pave the way for multiscale modelling strategies: the computed onset superheats and spatial statistics of bubble nucleation (captured, e.g., by a random Poisson point process) can be directly passed to macroscopic solvers. This opens the long-sought route to bridging fluctuation-driven nucleation with continuum-scale boiling simulations.

Overall, the present results demonstrate that fluctuating hydrodynamics, combined with a diffuse-interface description, provides a consistent framework to capture the full nucleation pathway of fluids, bridging mesoscopic statistical thermodynamics and hydrodynamics within a unified description. While the present study focuses on regimes close to the critical point, where the approach is most naturally justified (Langer & Turski 1973), such thermodynamic conditions are not merely academic but are also relevant to engineering applications, e.g. in pressurised water reactors operating at reduced temperatures *T* ≈ 0.9 and reduced pressures *p* ≈ 0.8, as well as in cryogenic and high-pressure systems.

More broadly, the framework is expected to remain valid away from criticality when complemented by rare-event techniques to access otherwise inaccessible activation regimes. The coupling between rare events and hydrodynamics, however, is intrinsically challenging; initial steps in this direction have been undertaken by the present authors in the homogeneous case, showing encouraging agreement with experiments for boiling at lower reduced temperatures (Gallo et al. 2026). Although demonstrated here for an ideal Van der Waals-type fluid, the framework provides a natural starting point for extensions toward more realistic thermodynamic descriptions through the adoption of accurate equations of state (Wagner & Pruß 2002; Kunz & Wagner 2012). When combined with modern high-performance computing capabilities, this approach has the potential to enable direct simulations spanning mesoscopic scales and to progressively capture the full boiling physics, from nucleation to macroscopic bubble dynamics, opening new perspectives for the modelling of phase-change phenomena.

From a fundamental standpoint, our findings also motivate theoretical developments in non-equilibrium statistical mechanics. By framing boiling as a fluctuation-activated process mediated by hydrodynamics, this work points to new avenues for understanding phase transitions beyond the limits of classical equilibrium thermodynamics.

## Data Availability

The final data supporting the research are made publicly available in the open Zenodo repository of the E-Nucl project, reachable at https://doi.org/10.5281/zenodo.20325087.
